# Down Syndrome Screening among Pregnant Women Visiting the Department of Obstetrics and Gynaecology of a Tertiary Care Centre

**DOI:** 10.31729/jnma.8293

**Published:** 2023-10-31

**Authors:** Jyotshna Sharma, Meena Thapa, Ranjana Yadav, Dipty Shrestha

**Affiliations:** 1 Department of Obstetrics and Gynecology, Kathmandu Medical College and Teaching Hospital, Sinamangal, Kathmandu, Nepal

**Keywords:** *down syndrome*, *nuchal translucency*, *screening*

## Abstract

**Introduction:**

The screening of Down's syndrome by the measurement of serum markers using dual and quadruple tests in the second trimester is done among obstetric patients between 13 to 22 weeks of gestation. The test readings are signified in terms of low-risk or high-risk. The aim of this study was to find out the prevalence of Downs syndrome screening among pregnant women visiting the Department of Obstetrics and Gynaecology of a tertiary care centre.

**Methods:**

A descriptive cross-sectional study was conducted from 15 April 2022 to 15 December 2022 among patients visiting the Department of Obstetrics and Gynecology of a tertiary care centre. Ethical approval was taken from the Institutional Review Committee. Women with a singleton pregnancy who underwent dual and quadruple screening tests at 11 to 22 weeks of gestation were taken and analysed as per reports. Convenience sampling method was used. The point estimate was calculated at a 95% Confidence Interval.

**Results:**

Among 268 women, Down syndrome screening was done in 200 (74.63%) (69.42-79.84, 95% Confidence Interval). Among them, 168 (84%) had a low risk for Down syndrome, and 32 (16%) had a high risk for Down syndrome.

**Conclusions:**

The prevalence of Downs syndrome screening among pregnant women visiting the Department of Obstetrics and Gynecology of a tertiary care centre was found to be similar to other studies done in similar settings.

## INTRODUCTION

Down syndrome screening, conducted in the second trimester using dual and quadruple tests, plays a crucial role in obstetric care, providing risk assessments for conditions like Down's syndrome, trisomy 18, and neural tube defects.^1^ The American College of Obstetricians and Gynecologists (ACOG) recommends quadruple testing for pregnant women, incorporating serum markers such as alpha-fetoprotein (AFP), human chorionic gonadotropin (hCG), unconjugated estriol, and inhibin A.^2^ However, challenges, including irregular hospital visits and missed early ultrasound scans, necessitate alternative approaches.

Quadruple screening is ideally performed between 15 and 18 weeks of gestation but can extend to 22 weeks.^3^ Studies indicate that sequential screening in both the first and second trimesters detects more Down syndrome cases.^4^ Aberrations in maternal serum markers, such as decreased alpha-fetoprotein and estriol levels alongside elevated hCG, are associated with Down's syndrome.^5^

The objective of this study was to find out the prevalence of Down syndrome screening among pregnant women in the Department of Obstetrics and Gynecology of a tertiary care centre.

## METHODS

This descriptive cross-sectional study was conducted among pregnant women visiting the Department of Obstetrics and Gynecology of Kathmandu Medical College Teaching Hospital, Sinamangal, Kathmandu, Nepal. Data was collected from December 2021 to December 2022. Ethical approval was taken from the Institutional Review Committee (Reference number: 25022022/03). Informed consent was taken. The pregnant women with singleton pregnancies who sought medical attention at the outpatient Department of the hospital during the stipulated data collection period were included. A convenience sampling method was used. The sample size was calculated using the following formula:


$$\begin{array}{ll} \text{n} & ={\text{Z}}^{2}\times \frac{\text{p}\times \text{q}}{{\text{e}}^{\text{2}}}\\ & =1.96^{2}\times \frac{0.50\times 0.50}{0.07^{2}}\\ & =196 \end{array}$$

Where,

n = minimum required sample size
Z = 1.96 at 95% Confidence Interval (CI)
p = prevalence taken as 50% for maximum sample size calculation
q = 1-p
e = margin of error, 7%

The minimum required sample size was 196. However, the final sample size taken was 268.

A detailed history was taken from the patients visiting the Outpatient Department and counselled about the test. They were screened for chromosomal abnormalities in the first trimester by NB (nasal bone) NT (nuchal translucency) scan along with dual marker and anomaly scan along with Quadruple test in the second trimester were enrolled in the study. Based on the result the women were classified into high risk and low risk for Down syndrome. All the women with reports of high risk were further connected and advised of amniocentesis or non-invasive prenatal tests.

The study assessed various components of antenatal screening for Down syndrome including, measurements of nuchal translucency, pregnancy-associated plasma protein A (PAPP-A) and the free beta subunit of human chorionic gonadotropin (fphCG), maternal age, serum alpha-fetoprotein, total human chorionic gonadotropin (hCG), unconjugated estriol, and inhibin A by using PRISCA 5 software.^5,6^

Data was entered and analysed using IBM SPSS Statistics version 20.0. The point estimate was calculated at a 95% CI.

## RESULTS

Among 268 women, Down syndrome screening was done in 200 (74.63%) (69.42-79.84, 95% CI). Among them, 168 (84%) had a low risk for Down syndrome and 32 (16%) had a high risk for Down syndrome.

Among 32 high-risk patients, 28 (87.50%) had a high risk for Down syndrome. Among them 20 (62.50%) went for non-invasive prenatal tests (NIPT) or amniocentesis after counselling from which 12 (37.50%) came normal and 8 (25%) came abnormal. They were also associated with other congenital abnormalities so opted for termination of pregnancy. Among 12 (37.50%) women who didn't do NIPT or amniocentesis, 8 (25%) delivered a normal baby and 4 (12.50%) were terminated pregnancies diagnosed with other congenital disorders. Among 200 women, 95 (47.50%) patients belonged to the age group of 31-40 years (Table 1).

**Table 1:** Age wise distribution of pregnant women undergoing screening for Downs syndrome (n= 200).

Age (years)	n (%)
< 20	9 (4.50)
21-30	95 (47.50)
31-40	90 (45)
>40	6 (3)

A total of 110 (55%) patients were multigravida (Table 2).

**Table 2:** Distribution of pregnant women according to gestation (n= 200).

Grivada	n (%)
Primigravida	90 (45)
Multigravida	110 (55)

Among 200 women, 21 (10.50%) patients had a high risk for Down syndrome according to the dual markertest whereas, 41 (20.50%) had a high risk according to the quadruple test (Table 3).

**Table 3:** Dual marker and quadruple test result (n= 200).

Test	Result	n (%)
Dual test	High risk	21 (10.50)
	Low risk	127 (63.50)
Quadruple test	High risk	11 (5.50)
	Low risk	41 (20.50)

## DISCUSSION

The prevalence of Down syndrome screening in our study was found to be 74.63%. In another study done in the US, it was found that 67-72% of pregnancies received prenatal screening for Down's syndrome which is similar to our study.^1^ This similarity in prevalence suggests that the practice of Down syndrome screening is relatively consistent across different healthcare settings, despite differences in healthcare systems and resources.

Down syndrome screening plays a crucial role in prenatal care, as it allows for the identification of pregnancies at increased risk for this genetic condition.^7^ According to the sources mentioned, Down syndrome screening has become an integral part of antenatal care in many developed countries. Maternal serum screening, which is based on biochemical markers present in maternal serum during the second trimester of pregnancy, has been established as an effective method for detecting Down syndrome.^8^ This screening method has been shown to result in a decrease in the incidence of Down syndrome in developed countries. However, the prevalence of Down syndrome in countries with low-resource settings has not seen significant changes in recent years.^9^

The use of amniocentesis as a classical practice for screening for Down's syndrome in the past suggests that any elderly pregnant women should receive screening for the presence of Down syndrome in the foetus. In addition, the ACOG has recommended that all pregnant women be offered prenatal screening for Down syndrome, regardless of age.^7^ The recommendation by the ACOG reflects the evolving understanding of Down syndrome screening and the importance of early detection.^2^

The recommendation to offer prenatal screening for Down syndrome to all pregnant women, regardless of age, is a significant shift in practice. This recommendation acknowledges that advanced maternal age is no longer the sole criteria for offering Down syndrome screening. Furthermore, offering routine amniocentesis to women aged 35 years or older without first performing maternal serum screening is now considered outdated. The adoption of maternal serum screening for Down syndrome in younger women has contributed to a marked decrease in Down syndrome live births in certain countries, such as Taiwan.^7^

As a matter of fact, the ACOG recommends that quadruple testing to be offered to pregnant women.^2^ The serum markers include a-fetoprotein, human chorionic gonadotropin, unconjugated estriol and inhibin A. In our context, patients come to the hospital irregularly and might miss the ultrasonography at 12-13 weeks which is an appropriate time to measure nuchal translucency and nasal bone and dual marker so a quadruple test with anomaly scan is advised. The quadruple screening tests are ideally done between 15 and 18 weeks of gestation but can be done up to 22 weeks. For any pregnant lady whose risk is low (no positive family history, etc.), the screening test provides reassurance that there is a decreased chance for Down's syndrome, trisomy 18, and neural tube defects. For those women in whom the quadruple test indicates a risk of any of the aforementioned conditions, additional screening tests should be considered.^3^

Between first-trimester and second-trimester screening, studies have shown that sequential screening in both the first and second trimester detects a greater number of Down syndrome babies.^4^ Low levels of maternal serum alpha-fetoprotein (MSAFP) and estriol and high hCG has been shown to be associated with Down's syndrome. Alternatively, lower levels of all of these markers are associated with Edward's syndrome.^5^ Studies have also shown that these quadruple markers could also be used in the prediction of small for gestational age babies.^10^ Studies suggest that the maternal serum AFP and unconjugated estriol are reduced on average by 2530% in pregnancies with Down's syndrome,^11-13^ and the levels of hCG and inhibin A are twice as high as those in normal pregnancies.^14-15^ Prenatal screening for specific foetal abnormalities has become a part of routine obstetrical care.^16^

While our study provides valuable insights into the prevalence of Down syndrome screening in a specific healthcare setting, it is essential to recognize that the findings may not be directly applicable to other regions or healthcare facilities. The generalizability of our results is limited by the single-centre design and the specific population studied. To obtain a more comprehensive understanding of the prevalence of Down syndrome screening, further research involving larger and more diverse populations is warranted.

This study has some limitations that should be considered when interpreting the results. Firstly, this study was conducted as a single-centre which limits the generalizability (external validity) of the findings to a broader population.

## CONCLUSIONS

The prevalence of Downs syndrome screening among pregnant women visiting the Department of Obstetric and Gynaecology of a tertiary care centre was found to be similar to other studies done in similar settings. Screening of Downs syndrome is recommended to improve the well-being of both mothers and foetuses through early detection and intervention of such genetic condition.
